# Generation of humanized TREM2 and hTREM2‐R47H mouse lines and their effects on disease pathogenesis in 5xFAD mice

**DOI:** 10.1002/alz70855_103726

**Published:** 2025-12-24

**Authors:** Min Sung Gee, Shimako Kawauchi, Giedre Milinkeviciute, Christiana Prekopa, Masashi Kitazawa, Andrea J Tenner, Frank M LaFerla, Grant R MacGregor, Kim N. Green

**Affiliations:** ^1^ University of California, Irvine, Irvine, CA, USA

## Abstract

**Background:**

The TREM2 R47H (rs75932628) coding variant has been identified as one of the most strongly associated genetic risk factors for late‐onset Alzheimer's disease (LOAD). Previous studies revealed that this missense mutation causes microglia to be less reactive to the amyloid beta (Aβ) plaques, which might exacerbate the progression of Alzheimer's disease (AD) pathologies. In an effort to develop mouse models for LOAD, we have generated human TREM2 (hTREM2) knock‐in and hTREM2‐R47H knock‐in mouse lines, fully replacing the murine *Trem2* (mTrem2) sequence with its human *TREM2* counterpart between start and stop codons, and crossed them with 5xFAD mouse line, a commonly used AD mouse model with robust amyloid‐beta pathology. We anticipate that these mouse models will shed light on the impact of microglial TREM2‐R47H variant in AD pathology development.

**Method:**

6 genotype groups were developed (WT, WT;hTREM2, WT;hTREM2‐R47H, 5xFAD, 5xFAD;hTREM2, 5xFAD;hTREM2‐R47H) with two age time points (4mo, 12mo). Mouse brains were isolated and cut in half; one hemisphere for immunohistochemistry (IHC) and the other for spatial transcriptomic analysis. Amyloid pathology and glial responses were measured via IHC. In spatial transcriptomic analysis using CosMx, transcriptomic characteristics and changes in each glial cell type were analyzed.

**Result:**

At 4 months of age, 5xFAD mice with the hTREM2‐R47H variant showed increased amyloid plaque load in subiculum, and a significant spread of amyloid plaque burden in cortex area relative to both 5xFAD with murine *Trem2*, as well as with hTREM2. Microglia also failed to respond to plaques. CosMx results indicated that hTREM2‐R47H knock‐in microglia failed to downregulate homeostatic gene expression (i.e., *P2ry12, Csf1r, Tgfbr1, and Tmem119)* compared to hTREM2 knock‐in line.

**Conclusion:**

Microglial reactivity to amyloid plaques was less in hTREM2 knock‐in line compared to mTrem2, suggesting that hTREM2 itself is not fully functional in the murine biologic system. Nevertheless, compared to mTrem2 and hTREM2, hTREM2‐R47H microglia do not down‐regulate their homeostatic genes in response to amyloid‐beta. Further, the magnitude of the effect of R47H in hTREM2 appears stronger than R47H in mTREM2, suggesting that humanization of disease relevant genes offers additional insights into disease mechanisms.

